# Supplementary material for: Head-to-head comparison between plasma p-tau217 and flortaucipir-PET in amyloid-positive patients with cognitive impairment
Source: Alzheimers Res Ther. 2023 Sep 22;15:157. doi: 10.1186/s13195-023-01302-w (PMC10517500; doi:10.1186/s13195-023-01302-w)
Supplement: Supplementary file 1 — Additional file 1: Supplementary Figure 1. Study flow-chart. Supplementary Table 1. Effect of Sex on the associations of plasma p-tau217 and FTP-PET with demographics and other variables. Supplementary Table 2. Comparison of the associations of plasma p-tau217 and FTP-PET with other variables using bootstrapping. Supplementary Table 3. Associations of plasma p-tau217 and FTP-PET with demographics and other variables after log-transformation or based on rank-based statistics. Supplementary Table 4. Cross-sectional models explaining MMSE scores using tau biomarkers and years of education. Supplementary Methods: R code. [file 13195_2023_1302_MOESM1_ESM.docx]

**Supplementary Materials**

**Supplementary Figure 1. Study flow-chart**

No PIB-PET scans available; *n* = 7

PIB-PET positive cases: *n* = 100

Plasma specimen with tau-PET; *n* = 186

PIB-PET negative cases; *n* = 79

PIB-PET: *n* = 179

AD spectrum diagnosis; *n* = 88

Non-AD clinical diagnoses;

*n = 11 (CBD, CN, FTD, PPA, TBI)*

*n = 1 (GRN mutation)*

UCSF patients with plasma data; *n* = 477

No tau-PET scans available; *n* = 291

tauPET $\pm$ 365 days from plasma

*n = 1*

***n = 87***

**Supplementary Table 1. Effect of Sex on the associations of plasma p-tau217 and FTP-PET with demographics and other variables.**

**
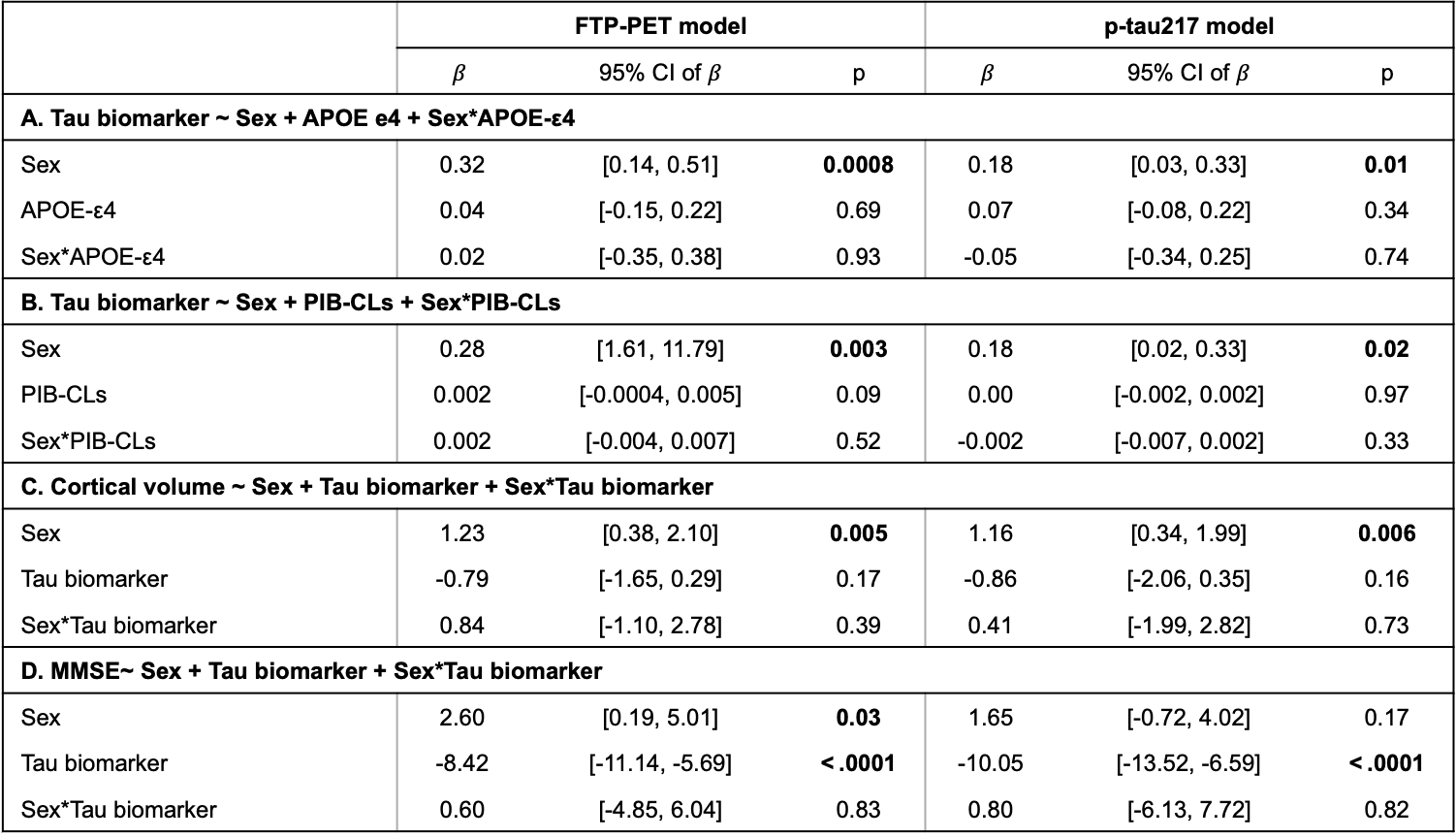
**

*Models A-C include all 87 patients, while Model D includes 85 patients. Sex is dummy coded as 0 for Males and 1 for Females so positive estimates indicate higher tau biomarker/volumes/MMSE scores in Females. APOE-*ε4 *is coded as 0 for non-carrier and 1 for carrier so positive estimates indicate higher tau biomarker/volumes/MMSE scores in APOE-*ε4 *carriers. Other variables are centered, not standardized.*

**Supplementary Table 2. Comparison of the associations of plasma p-tau217 and FTP-PET with other variables using bootstrapping.**

|  | **Age** | | **Sex** | | **APOE (number of ε4 alleles)** | | **Amyloid-PET (CLs)** | |
| --- | --- | --- | --- | --- | --- | --- | --- | --- |
|  | r | p | d | p | $\eta^{2}$ | p | r | p |
| FTP-PET | -0.68 | <0.001 | 0.78 | <0.001 | 0.002 | 0.91 | 0.26 | 0.02 |
| p-tau217 | -0.44 | <0.001 | 0.53 | <0.001 | 0.015 | 0.52 | 0.10 | 0.36 |
|  | $\Delta r$ [95%CI] | p | $\Delta d$ [95%CI] | p | $\Delta\eta^{2}$ [95%CI] | p | $\Delta r$ [95%CI] | p |
| Bootstrap | -0.24  [-0.40, -0.08] | 0.003 | 0.25  [-0.16, 0.72] | 0.21 | -0.013  [-0.08, 0.06] | 0.81 | 0.16  [-0.01, 0.34] | 0.09 |

R code used for these analyses is available at the bottom of this supplementary material document.

**Supplementary Table 3. Associations of plasma p-tau217 and FTP-PET with demographics and other variables after log-transformation or based on rank-based statistics.**

| **Variables** | | **Raw** | | **Log-transformed** | | **Non-parametric** | |
| --- | --- | --- | --- | --- | --- | --- | --- |
| FTP – p-tau217 | | r | 0.61 | r | 0.66 | $\rho$ | 0.64 |
|  |  | p | <.001 | p | <.001 | p | <.001 |
| Age | FTP | r | -0.68 | r | -0.67 | $\rho$ | -0.68 |
|  |  | p | <.001 | p | <.001 | p | <.001 |
|  | p-tau217 | r | -0.44 | r | -0.46 | $\rho$ | -0.48 |
|  |  | p | <.001 | p | <.001 | p | <.001 |
| Sex | FTP | d | 0.78 | d | 0.80 | rrb | 0.39 |
|  |  | p | <.001 | p | <.001 | p | <.001 |
|  | p-tau217 | d | 0.53 | d | 0.56 | rrb | 0.29 |
|  |  | p | 0.016 | p | 0.01 | p | 0.02 |
| APOE-ε4  (number ε4 alleles) | FTP | $\eta^{2}$ | 0.002 | $\eta^{2}$ | 0.001 | $\varepsilon^{2}$ | 0.002 |
|  |  | p | 0.91 | p | 0.95 | p | 0.92 |
|  | p-tau217 | $\eta^{2}$ | 0.015 | $\eta^{2}$ | 0.007 | $\varepsilon^{2}$ | 0.003 |
|  |  | p | 0.52 | p | 0.74 | p | 0.87 |
| PIB CLs | FTP | r | 0.26 | r | 0.26 | $\rho$ | 0.19 |
|  |  | p | 0.02 | p | 0.015 | p | 0.08 |
|  | p-tau217 | r | 0.10 | r | 0.21 | $\rho$ | 0.07 |
|  |  | p | 0.36 | p | 0.057 | p | 0.52 |
| MMSE | FTP | r | -0.530 | r | -0.530 | $\rho$ | -0.509 |
|  |  | p | <.001 | p | <.001 | p | <.001 |
|  | p-tau217 | r | -0.525 | r | -0.507 | $\rho$ | -0.443 |
|  |  | p | <.001 | p | <.001 | p | <.001 |

*For log-transformed associations, only FTP-SUVR and p-tau217 concentrations were log-transformed; other all values remained untransformed. For non-parametric measures, spearman’s test was used for continuous variables (*$\rho$ *= Spearman’s rho (rank-based)) coefficient), Mann-Whitney U test for binary variables (rrb = rank biserial r), Kruskal-Wallis ANOVA for categorical variables with more than 2 categories (*$\varepsilon^{2}$*: effect size from Kruskal-Wallis non-parametric ANOVA)*

**Supplementary Table 4. Cross-sectional models explaining MMSE scores using tau biomarkers and years of education.**

***
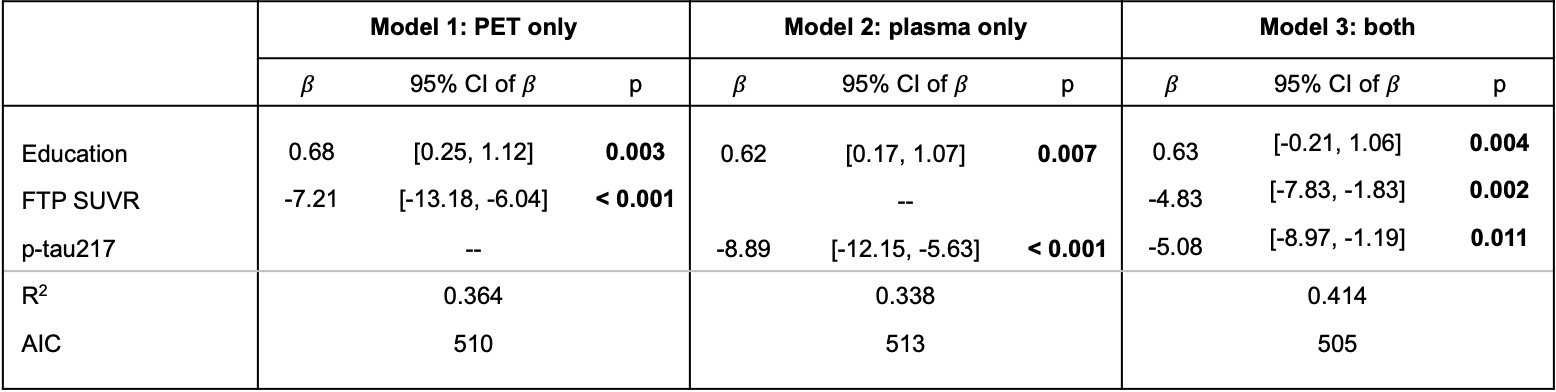
***

*All models use MMSE score as the dependent variable and include 83 patients (n=4 missing info on years of education; 2/4 missing MMSE as well). Bivariate correlation between MMSE and years of education: r = 0.31, p = 0.0047.*

*R^2^ = Coefficient of Determination (higher R^2^ is better), AIC = Akaike Information Criterion (lower AIC is better).*

**Supplementary Methods: R code**

**Code for bootstrapped resampling**

Example R code below showing difference in corresponding effect sizes between tau-PET and age and p-tau217 and age:

library(cocor)

library(boot)

library(boot.pval)

data_age <- data.frame(age=bldf$bl_age, pet=bldf$bl_FTP, plasma=bldf$bl_pTau)

boot.function <- function(data_age,indices

data_age <- data_age[indices,]

corr1 <- cor.test(data_age$age,data_age$pet)

corr2 <- cor.test(data_age$age,data_age$plasma)

diff <- corr1$estimate-corr2$estimate

return(diff)

}

set.seed(12345)

boot.out <- boot(data_age,boot.function,R=1000)

# CI

boot.ci(boot.out,type="perc")

# P-value

boot.pval(boot.out,type="perc")

The above code was repeated similarly for amyloid-PET Centiloids; however, the difference in effect sizes was calculated cohen’s d for sex and eta-squared for APOE- ε4.

**Code for linear mixed effect models**

library(lme4)

library(lmerTest)

library(nlme)

library(dplyr)

df <- read.table("05-Longitudinal_MMSE_NM_06-25.csv", header = TRUE, sep = ",")

mmsedf_lme <- df[c(1,4,7,8)]

colnames(mmsedf_lme) <- c("ID","time_from_first_mmse","bl_FTP","bl_pTau")

### Mean Center function

len <- length(colnames(mmsedf_lme))

center_scale <- function(x) {

scale(x, scale = FALSE)

}

# Select columns that don’t need centering

mmsedf_lme_nocentering <- mmsedf_lme[,1]

# Select columns that need centering

mmsedf_lme_centering <- center_scale(select_if(mmsedf_lme[,3:len], is.numeric))

# Bind non-mean centered data and mean centered data into one dataset

mmsedf_lme_centered <- cbind(mmsedf_lme_nocentering, mmsedf_lme_centering)

# Model 1: LME model with FTP

longitmmse_FTP <- lmer(MMSE ~ 1 + time_from_first + bl_FTP + time_from_first*bl_FTP + (1 | ID), data = mmsedf_lme_centered)

summary(longitmmse_FTP)

# Model 2: LME model with p-tau217

longitmmse_pTau <- lmer(MMSE ~ 1 + time_from_first + bl_pTau + time_from_first*bl_pTau + (1 | ID), data = mmsedf_lme_centered)

summary(longitmmse_pTau)

# Model 3: LME model with FTP and p-tau217

longitmmse_pTauFTP <- lmer(MMSE ~ 1 + time_from_first + bl_pTau +bl_FTP + time_from_first*bl_pTau + time_from_first*bl_FTP + (1 | ID), data = mmsedf_lme_centered)

summary(longitmmse_pTauFTP)
